# Beyond the Classic Causes of Dysphagia: Bayford-Autenrieth Dysphagia

**DOI:** 10.7759/cureus.54755

**Published:** 2024-02-23

**Authors:** Luis Manuel Sáenz, Raul Eduardo Quintero Castro, Abraham Enrique Herrera Torres, Miriel Orella Castro, Luis Andres González-Torres

**Affiliations:** 1 Internal Medicine Department, Hospital Universitario Dr. José Eleuterio González, Monterrey, MEX

**Keywords:** acute dysphagia, bayford-autenrieth dysphagia, vascular dyspaghia, dyspaghia lusoria, dysphagia

## Abstract

Dysphagia lusoria (DL) is a rare clinical entity that presents with dysphagia derived from the anatomical obstruction of the esophagus by an aberrant vessel originating from the right subclavian artery. We present the case of a 64-year-old patient with a medical history of chronic, intermittent, mild, and self-limited dysphagia for over 20 years, wherein we formulated the diagnosis of DL. A 64-year-old woman arrived at the emergency department with a 24-hour history of acute progressive dysphagia, leading to intolerance to oral intake and minimal exertion dyspnea. A thorough clinical analysis and exclusion of other more common clinical entities will lead to its diagnosis. Our patient presented with respiratory symptoms, which is rare considering that these clinical presentations are more common in the pediatric population, explained by its tracheal elasticity. The combination of respiratory symptoms in an elderly patient, along with the typical mechanical dysphagia of DL, adds complexity to the diagnostic process, making this case unique.

## Introduction

Dysphagia lusoria (DL) refers to the vascular compression of the esophagus caused by an aberrant right subclavian artery (ARSA), originating from the left side of the aortic arch. This anatomical variant is the most common of the aortic arch and has a 1.2%-2.2% prevalence in the general population. ARSA is asymptomatic in 90% of cases, and it is typically incidentally discovered during imaging studies. While symptomatology is infrequent, dysphagia serves as the primary presenting symptom. Achalasia and gastrointestinal neoplasms are common initial differential diagnoses before considering DL as the primary diagnosis. DL represents a potential diagnosis when endoscopic and radiological examinations do not provide clarity in cases of atypical dysphagia. 

The term "Bayford-Autenrieth dysphagia" originates from David Bayford's 1787 report, detailing the first documented case of dysphagia characterized by fatal obstructed deglutition. This nomenclature acknowledges Bayford's significant contribution to understanding dysphagia, with Autenrieth potentially playing a role in subsequent advancements in the field [1].

The lusoria artery is more frequently observed in women and associated with other conditions such as Down syndrome, DiGeorge syndrome, and Edwards syndrome as they carry a higher risk of vascular malformations. Age is a determinant of type of DL clinical manifestations; pediatric patients typically exhibit respiratory symptoms such as wheezing, stridor, dyspnea, and pneumonia, while adults tend to experience chronic dysphagia. Tracheal elasticity may explain these findings [1,2]. We present a unique case of an adult DL patient with respiratory symptoms.

## Case presentation

**Figure 1:**
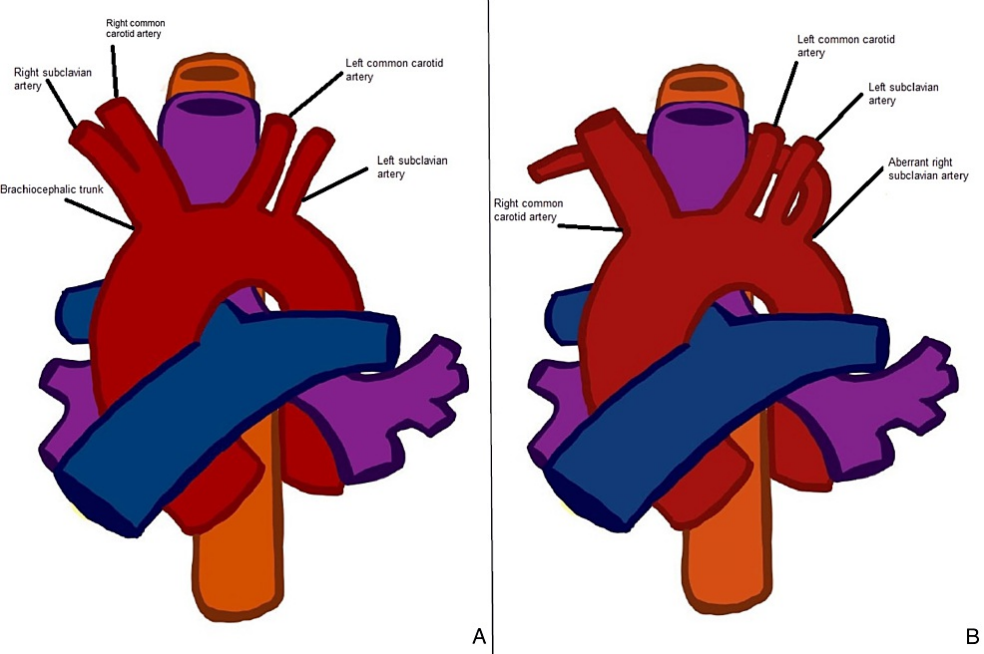
This culprit artery is considered "aberrant" as it deviates from the norm; typically, there are only three vessels originating from the aortic arch (A). In the case of an ARSA, the aortic arch is composed of four vessels instead of three (B). Schematic diagram of the aortic arch with an ARSA

**Figure 2:**
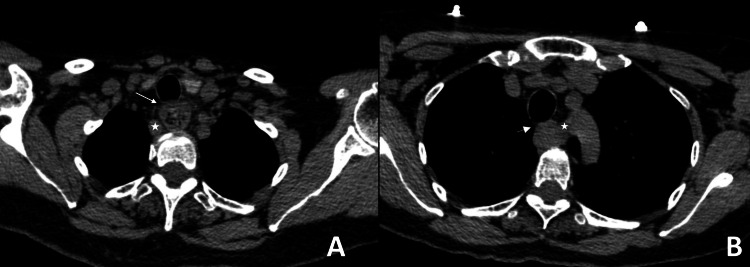
Distal compression of the esophagus by an aberrant vessel (star), which, in turn, causes dilation of the esophagus with remnants of food observed inside and tracheal compression (arrow) (A). Culprit artery originating from the aortic arch following a path toward the right (star) and collapsed esophagus due to vascular compression, also compressing the trachea (arrow) (B). Contrast-enhanced CT scan of the neck

A 64-year-old female with a medical history of chronic dysphagia and a smoking history of 22 packs per year initiates her clinical complaint 24 hours before her emergency department (ED) admission with acute dysphagia, coughing, and respiratory stridor. She seeks care at an outpatient clinic where a neck X-ray reveals radiolucency in the supraglottic region. She reports experiencing similar episodes for over 20 years without recent exacerbations, described as mild, intermittent, short-lasting, sporadic, and self-limiting. Before, healthcare professionals refrained from assessing these episodes using imaging tools. She described this new onset as the “worst ever.” Worsening dysphagia with oral intake compromise and minimal exertional dyspnea caused her to seek medical management in the ED. The physical examination showed a blood pressure of 120/80, heart rate of 102 beats per minute (bpm), respiratory rate of 24, temperature of 36.7°C, and pulse oximeter saturation of 92%. We detected tracheal stridor upon auscultation. Chest rays revealed esophageal dilatation with probable distal obstruction. A CT scan of the neck revealed the presence of an ARSA originating from the left side of the aortic arch crossing over to the right side, causing compression of the esophagus from behind and providing an explanation for the respiratory symptoms (Figures 1A-1B, 2A-2B).

The radiology department recommended performing a CT angiography of the supra-aortic vessels to determine the correct management. We conducted a targeted inquiry to rule out a history of chronic gastroesophageal reflux or medication abuse (nonsteroidal anti-inflammatory drugs, NSAIDs) to rule out esophageal peptic stenosis. Gastroenterology consultation resulted in the patient's admission for further evaluation and endoscopy. Upper endoscopy and esophageal manometry ruled out mechanical obstruction and achalasia.

After 48 hours, the patient reported the typical clinical resolution of her symptoms (an intermittent and resolving condition she usually experiences) and communicated her desire for voluntary discharge and outpatient care.

## Discussion

We have identified some key strengths and limitations in our case study. On the positive side, we have observed an unusual clinical presentation of acute dysphagia in an adult patient with respiratory symptoms. Additionally, we have conducted a thorough analysis of the existing literature on respiratory clinical presentations of DL in adults, and we have used advanced diagnostic tools such as CT scans, endoscopies, and manometries to rule out other potential diagnoses. However, there are also some limitations to our study. First, we were unable to perform a 3D reconstruction, which would have been useful to observe the vascular anatomy. Second, we were only able to conduct an early discharge, and, therefore, we were unable to follow up on the effectiveness of the treatment we administered.

An ARSA was first described in 1735 by the French anatomist François-Joseph Hunauld. Years later, in 1787, David Bayford first described DL in a female patient with a medical history of lifelong dysphagia [1]. The culprit artery follows a retroesophageal path in 80% of cases, 15% between the trachea and esophagus, and 5% anterior to the trachea [3]. In a typical scenario, three main vessels branch off the aortic arch: the brachiocephalic trunk, the left common carotid artery, and the left subclavian artery. Additionally, the right common carotid artery and the right subclavian artery originate from the brachiocephalic trunk. However, in the presence of the ARSA, the brachiocephalic trunk is absent.

Consequently, four major vessels will arise from the aortic arch: the right common carotid artery, the left common carotid artery, the left subclavian artery, and the right subclavian artery, also referred to as the arteria lusoria. This anomalous artery extends toward the right arm, crossing the midline, with the Kommerell diverticulum being this artery's most common origin point [1,4]. Our patient presented with a typical retro-esophageal ARSA.

Decreased elasticity of the esophagus, increased rigidity of the trachea, vascular changes resulting from atherosclerosis that lead to artery hardening, aortic elongation causing greater traction on the artery, and aneurysmal dilation in the presence of a Kommerell diverticulum represent the main factors to late-onset symptoms [5].

Manifestations of this condition vary depending on the age group. Pediatric patients typically exhibit respiratory symptoms such as wheezing, stridor, dyspnea, and pneumonia, while adults experience chronic dysphagia. Vascular compression of the esophagus results in dysphagia, and tracheal compression results in respiratory symptoms that may resemble infectious conditions, pneumonia, pseudo-asthma, and stridor, which is uncommon in adults. The tracheal elasticity of the children results in easier compression, thus increasing respiratory symptomatology. Dysphagia (71.2%), dyspnea (18.7%), retrosternal pain (17.0%), cough (7.6%), and weight loss (5.9%) are the most commonly reported symptoms. Notably, there is a significant difference between men and women in terms of the average age of onset, with women experiencing symptoms at an average age of 44.9 years, compared to men at an average age of 54.0 years. Women's estrogen reduction with accelerated atherosclerotic changes may explain this finding [1]. Our patient presented with acute dysphagia and respiratory symptoms, making it a rare occurrence in an adult patient.

Previously reported cases have described atypical respiratory presentations in adults, such as pseudo-asthma, de novo dyspneic episodes, and aspiration pneumonia (Table 1) [6-12].

**Table 1:** Similar case reports in the literature.

Publication year	Author	Relevant findings
2023	Mawait et al. [6]	A 50-year-old woman presented with a history of chronic dysphagia and dyspnea during meals, resulting in oral intake compromise and a 3 kg weight loss in a few months. The patient had several risk factors for atherosclerotic disease and a history of hypertension, chronic obstructive pulmonary disease (COPD), and dyslipidemia.
2020	Massaro et al. [7]	An 83-year-old woman was diagnosed with acute myeloid leukemia and COPD. The patient presented with dyspnea due to exacerbation of COPD, bilateral pleural effusion, and mechanical dysphagia for solids. Eighteen days after admission, the patient developed a sudden progressive dysphagia and dyspnea. She required intubation and ICU management. The patient died 24 hours later because of acute respiratory failure.
2018	Spatenkova et al. [8]	A 67-year-old patient with a medical history of pre-stroke and hypertension, diagnosed with a minor left intracerebral hemorrhage in the left basal ganglia manifested sudden dyspnea and a decrease in SpO2 with multiple X-ray findings that suggested aspiration pneumonia that developed into acute respiratory failure.
2016	Chudasama et al. [9]	An 81-year-old patient with a history of coronary artery disease, atrial fibrillation, coronary stent placement, diabetes mellitus, hypertension, dyslipidemia, urinary incontinence, and a treated non-Hodgkin’s lymphoma of the right thigh presented with dyspnea on exertion that started nine months before admission, underwent aortic valve replacement and after the surgery, and developed progressive dysphagia to solids and liquids, including saliva. Their other symptoms included regurgitation of undigested food.
2009	Dandelooy et al. [10]	A 76-year-old woman presented with several months of difficulty swallowing and shortness of breath; she had no known comorbidities or risk factors.
2002	Morel et al. [11]	Two cases of diverticulum of Kommerell wherein tracheal compression induced respiratory symptoms mimicking asthma. Case 1 reports a 28-year-old patient in the third month of her fourth pregnancy because of increasing dyspnea, with a history of exertion dyspnea that began several years before. The physical examination was unremarkable, and the patient had no known comorbidities or risk factors. Case 2 presents a 31-year-old patient with a history of mild asthma since childhood who sought medical attention for a worsening of her symptoms despite increasing bronchodilator therapy. Her pulmonary tests showed moderate obstruction patterns with response to ß-adrenergic stimulation. The patient did not have any additional comorbidities or risk factors.
2001	Störk et al. [12]	In this article, the authors discuss a 79-year-old woman with dysphagia and dyspnea and explore the relationship between DL symptoms and coexisting coronary artery disease.

Our case could help broaden the clinical spectrum known for DL in adults. Previously reported cases have noted an association between DL and increased cardiovascular risk factors, which may contribute to atherosclerotic arterial remodeling. This arterial remodeling can lead to greater rigidity and compression force in the culprit artery, ultimately resulting in obstructive symptoms [6,9]. Our patient had important atherosclerotic risk factors, such as age, obesity (BMI: 31 kg/m^2^), a sedentary lifestyle, and significant history of smoking.

Sudden dysphagia is a worrisome symptom in adult patients and yields the exclusion of neoplastic causes [13]. Our patient had a relevant history of smoking with no other neoplastic risk factors. The upper endoscopy ruled out oncological pathology.

Differential diagnoses include eosinophilic esophagitis, peptic strictures, primary achalasia, motility disorders, and scleroderma [14,15]. We ruled out these as the patient had no history of chronic gastroesophageal reflux or drug abuse (NSAIDs), and the endoscopic and manometric results revealed no significant findings. Blood count findings such as chronic iron deficiency anemia may suggest the presence of esophageal webs (Plummer-Vinson syndrome) as the primary cause of dysphagia; our patient did not present laboratory abnormalities. Additionally, our patient did not have a history of recent chest, head, or neck radiation therapy and did not ingest caustic substances, ruling out chemical esophagitis and postradiation esophageal stenosis [16].

Diagnosis involves a thorough physical examination (asymmetrical radial pulses) and radiological, endoscopic, and manometric methods. Upper endoscopy findings may describe a pulsating extrinsic compression of the esophagus, but the prevalence of this finding is rare. Esophageal manometry often reveals a high-pressure zone proximal to the narrowed site and elevated peristaltic pressures above the compressed area. Esophagogram, followed by CT and MRI scans, represents the most effective method for diagnosing DL. Angiography has no role in DL diagnosis [17].

The management of the condition depends on the severity of symptoms in each patient. For those with mild to moderate symptoms, the strongest recommendation is to make lifestyle modifications and dietary changes, including aggravating food avoidance, slow-paced eating, smaller bites, and limiting liquid intake in addition to acid suppressants and promotility agents [17]. There is no established standard treatment for DL.

Our patient's case highlights the importance of treating atherosclerotic risk factors, which can cause vascular changes and arterial stiffness [5].

If conservative treatments fail to provide relief, surgery may represent a therapeutical option. The surgical technique will depend on the patient's vascular anatomy and the surgeon's preference. The primary aim of the surgical procedure is to correct the abnormal position and functionality of the affected vessel. The conventional method involves dividing the affected artery at its origin through a median sternotomy and relocating the subclavian artery. A cervical approach grants better visibility [18]. Endoscopic dilation of the narrowed esophagus or interventional radiology procedures are alternative therapeutic options for patients who do not qualify for surgery or who have refractory symptoms [19,20]. Our patient does not qualify as an ideal candidate for surgical intervention, considering age and surgical risk factors. Lifestyle changes and atherosclerotic factor attendance convey the appropriate treatment at the moment. However, this does not exclude a potential benefit in the future if we observe clinical persistence.

## Conclusions

The compression of the esophagus due to a rare anatomical variation of the aortic arch is an uncommon cause of dysphagia and dyspnea in adults. The first step in diagnosis is ruling out more common and concerning pathologies.

Our patient's detailed description of chronic dysphagia represented a diagnostic challenge; long-term follow-up details would have complemented our case report. The cardiovascular risk factors contribute to arterial remodeling, resulting in heightened arterial stiffness, compression force, and respiratory symptoms. Understanding this relationship may offer a therapeutic avenue for addressing atherosclerotic risk factors in these patients. Presenting this case is essential for enhancing knowledge of the diverse clinical presentations of this rare condition.
